# Baicalein facilitates gastric cancer cell apoptosis by triggering endoplasmic reticulum stress via repression of the PI3K/AKT pathway

**DOI:** 10.1186/s13765-022-00759-x

**Published:** 2023-02-13

**Authors:** Junjie Shen, Zhiwen Yang, Xinlin Wu, Guodong Yao, Mingxing Hou

**Affiliations:** 1 Nanjing University of Chinese Medicine, Nanjing, 210029 Jiangsu province China; 2 Gastrointestinal Surgery, Affiliated Hospital of Inner Mongolia Medical University, Hohhot, 010050 Inner Mongolia China; 3 The Affiliated Hospital of Inner Mongolia Medical University, No. 1, Datong North Street, Huimin District, 010050 Hohhot, Inner Mongolia China

**Keywords:** Baicalein, Gastric cancer, PI3K/AKT, Endoplasmic reticulum stress, BTG3, Cell apoptosis, Ca^2+^, Ki67

## Abstract

**Objective:**

Gastric cancer (GC) remains a prevailing threat to life. Baicalein exhibits anti-cancer properties. This study estimated the mechanism of baicalein in GC cell apoptosis by mediating endoplasmic reticulum stress (ERS) through the PI3K/AKT pathway.

**Methods:**

After treatment with different concentrations of baicalein, GC cell (HGC-27 and AGS) viability was detected by MTT assay. AGS cells more sensitive to baicalein treatment were selected as study subjects. The IC50 of baicalein on AGS cells was determined. Colony formation, cell cycle, and apoptosis were detected using crystal violet staining and flow cytometry. Levels of ERS-related and BTG3/PI3K/AKT pathway-related proteins were determined by Western blot. Intracellular Ca^2+^ level was measured using Fluo-3 AM fluorescence working solution. GC mouse models were established by subcutaneously injecting AGS cells into the right rib and were intragastrically administrated with baicalein. Tumor volume and weight were recorded. Expression of Ki67 in tumor tissues and positive expression of apoptotic cells were detected by immunohistochemistry and TUNEL staining.

**Results:**

Baicalein inhibited cell proliferation and induced G0/G1 arrest and apoptosis by regulating the cell cycle, and triggered ERS in GC cells. Baicalein impeded the PI3K/AKT pathway by activating BTG3, thereby triggering ERS and inducing apoptosis. BTG3 inhibition reversed baicalein-induced apoptosis and ERS. Baicalein regulated GC cells in a concentration-dependent manner. Moreover, in xenograft mice, baicalein prevented tumor growth, decreased Ki67-positive cells, activated BTG3, and inhibited the PI3K/AKT pathway, thus activating ERS and increasing apoptotic cells.

**Conclusion:**

Baicalein facilitates GC cell apoptosis by triggering ERS via repression of the PI3K/AKT pathway.

## Introduction

Gastric cancer (GC) ranks the 5th most prevailing cancer globally, which accounts for approximately 1 million new cases and above 720 000 deaths annually [1]. Most human GC occurs following long-term Helicobacter pylori infection through the Correa pathway, involving the following processes: gastritis, atrophy, intestinal metaplasia, dysplasia, and finally cancer [2]. Individuals with newly diagnosed GC frequently present with dyspepsia and reflux, and the patients at the advanced stage may exhibit symptoms including weight loss, dysphagia, emesis, anemia, and gastrointestinal bleeding [3]. The common malnutrition occurring at advanced or metastatic GC can impact the life quality, enhance the toxicity of chemotherapy, and decrease overall survival [4]. Consequently, the 5-year survival for advanced GC individuals is only 5-20% [5]. Herein, it is extremely paramount to explore new valid treatments and medicines for GC.

Baicalein, a bioactive constituent found in *O. indicum* medicinal plant, exhibits numerous biological activities, including anti-cancer, anti-inflammatory, antibacterial, anti-adipogenesis, cardioprotective, neurogenesis, anti-hyperglycemia, and wound healing effects [6]. Baicalein is a representative flavonoid in Scutellaria baicalensis, and in modern clinical studies, Scutellaria baicalensis is widely adopted to treat an array of diseases, including acute respiratory infection, hypertension, acute gastroenteritis, trachoma hepatitis, infantile diarrhoea, and vomiting during pregnancy [7]. Shuang-Huang-Lian oral liquid, with Scutellaria baicalensis as one of the main ingredients, is considered a symptomatic treatment for coronavirus disease 2019 in China [8]. PHY906 containing Scutellaria baicalensis can enhance the therapeutic effect of anticancer drugs as the adjuvant to chemotherapy, with promising results examined in clinical studies for pancreatic, colorectal, and liver cancers [9]. Compelling evidence has vindicated the anti-cancer properties of baicalein by inducing cancer cell apoptosis through inhibition of phosphatidylinositol-3 kinase/protein kinase B (PI3K/AKT), such as breast cancer [10] and liver cancer [11]. Moreover, baicalein can enhance cisplatin sensitivity of GC cells by stimulating cell apoptosis and autophagy by affecting AKT/mTOR and Nrf2/Keap 1 pathways [12]. Although preceding reports have revealed the potential of baicalein in GC therapy, there still lacks systematic studies on the mechanism of baicalein in regulating GC.

The stimulation of internal pressure (including oncogenic activation) and external adverse environmental factors (such as hypoxia and nutritional deficiencies) that tumor cells are constantly subjected to may pose a serious threat to proteostasis [13]. Endoplasmic reticulum stress (ERS), a fundamental cellular stress response, sustains cellular protein homeostasis under exogenous or endogenous stimuli, which is also involved in the onset, progression, and drug resistance of tumors [14]. ERS response and the unfolded protein response (UPR) keep protein homeostasis in cells by increasing protein folding capacity and decreasing the intracellular load of secretory proteins or triggering cell apoptosis that cannot recover [15]. Furthermore, ERS determines the fate of cancer cells by modulating cell signaling networks during GC progression [16], identifying the substantial involvement of ERS in GC.

Although the function of ERS to induce apoptosis has been demonstrated by many researchers [17, 18], the underlying mechanism remains elusive. The PI3K/AKT pathway represents one of the most vital pathways that regulate cell proliferation, growth, and apoptosis in assorted cancers [19]. PI3K/AKT pathway also triggers stem cell-like properties in GC cells [20]. Besides, B-cell translocation gene 3 (BTG3) overexpression can block the PI3K/AKT/mTOR pathway activation [21], and BTG3 can regulate GC cell proliferation, migration, and apoptosis [22]. Recently, PI3K/AKT pathway is considered paramount in controlling cell survival by repressing ERS-induced cell apoptosis [23], so we speculated that PI3K/AKT pathway may also be implicated with the occurrence of ERS in GC cells. Therefore, this study probed into the regulation of baicalein in GC cell apoptosis by mediating ERS via the PI3K/AKT pathway.

## Materials and methods

### Ethics statement

All animal experiments were approved by the ethics committee of Affiliated Hospital of Inner Mongolia Medical University. Considerable efforts were conducted to minimize the animal number and their pain.

### Cell lines and culture

GC cell lines HGC-27 and AGS provided by Cell Bank of Type Culture Collection of Chinese Academy of Sciences (Shanghai, China) were cultured in RPMI-1640 medium containing 10% fetal bovine serum, 100 U/mL penicillin, and 100 µg/mL streptomycin at 37 °C.

### Drug

Baicalein provided by Sigma-Aldrich (Merck KGaA, Darmstadt, Germany) was dissolved in dimethyl sulphoxide (DMSO, Sigma-Aldrich) at 100 mmol/L as the stock solution and kept at − 20 °C in the dark. Baicalein was diluted in high-glucose Dulbecco’s modified Eagle medium to treat GC cells at indicated dosages.

### Cell viability assay

Drug sensitivity was estimated using the 3-(4,5-dimethylthiazol-2-yl)-2,5-diphenyltetrazolium bromide (MTT) assay. Briefly, HGC-27 or AGS cells were trypsinized and plated into 96-well plates (Corning, NY, USA) at 5 × 10^3^ cells per well. The cells were cultured overnight and next cultured with a fresh medium containing baicalein at different concentrations (0, 15, 30, 60, and 120 µM). After 48 h of incubation, 20 µL MTT (Sigma-Aldrich) was supplemented directly at the indicated times and dissolved in phosphate-buffered saline (PBS) at 5 mg/mL. Afterwards, the dishes were incubated for 4 h at 37 °C to conduct the MTT reaction. The supernatant was removed after 4 h. Subsequently, the formed formazan crystals were dissolved via addition of 100 µL DMSO, and the optical density (OD) at 490 nm was determined using a microplate reader (Bio-Tek, Norcross, GA, USA). Cell survival rate (%) = (OD of treatment group/OD of control group)* 100%.

### Cell transfection and grouping

AGS cells were selected to carry out in vitro studies since their cell viability was greatly affected by baicalein. Next, siRNA-negative control (NC) and siRNA-BTG3 (GenePharma, Shanghai, China) were respectively transfected into AGS cells (above 80% confluence) using the Lipofectamine 2000 (11668-019, Invitrogen, Carlsbad, CA, USA), and subsequent experiments were performed after 48 h of transfection.

Experiments were performed using different concentrations of baicalein or 4-PBA, LY294002 (all from Sigma-Aldrich), or DMSO (baicalein, 4-PBA, and Ly294002 were all dissolved in DMSO). AGS cells were treated and grouped as follows: blank group (without any treatment), DMSO group (treated with 0.1% DMSO, served as a solvent control group of baicalein group), Bai (15 µM) group (treated with 15 µM baicalein), Bai (30 µM) group (treated with 30 µM baicalein), Bai (60 µM) group (treated with 60 µM baicalein), Bai (30 µM) + DMSO group (treated with 30 µM baicalein and supplemented with solvent of 4-PBA, 0.1% DMSO), Bai (30 µM) + 4-PBA group (after treatment of 30 µM baicalein, added with 10 µM ERS inhibitor 4-PBA), Bai (30 µM) + siRNA-NC group (transfected with si-NC and then supplemented with 30 µM baicalein), Bai (30 µM) + siRNA-BTG3 group (delivered with si-BTG3 and later added with 30 µM baicalein), Bai (30 µM) + siRNA-BTG3 + DMSO group (after transfection with si-BTG3, supplemented with 30 µM baicalein and solvent of LY294002, 0.1% DMSO), and Bai (30 µM) + siRNA-BTG3 + LY294002 group (after transfection with si-BTG3, added with 30 µM baicalein and 10 µM PI3K/AKT pathway inhibitor LY294002). The doses of 4-PBA and LY294002 were determined according to the product instructions. The subsequent indicators were detected after 48 h of treatment.

### Colony formation assay

AGS cells were detached into single-cell suspension using trypsin-ethylene diamine tetraacetic acid (Gibco, Grand Island, NY, USA) solution. Subsequently, 2 mL cells were seeded onto 6-well plates (Corning) at 2 × 10^2^ cells/mL. After attachment, cells were treated with baicalein (0 and 30 µmol/L) for 48 h. The supernatant was substituted by fresh medium and subsequently cells were cultured for 15 days. Next, cells were fixed with 10% formalin and stained for 1 h with 0.1% crystal violet (Sigma-Aldrich). Thereafter, cells were rinsed and dried. Digital images were obtained using a microscope (Leica, Solms, Germany).

### Cell cycle analysis

The collected AGS cells were fixed overnight with 70% cold ethanol at − 20 °C. Next, after washing and resuspension in cold PBS, cells were incubated with 10 mg/mL RNase and 1 mg/mL propidium iodide (PI, Sigma-Aldrich) for 30 min at 37 °C. DNA content was measured utilizing the flow cytometer (BD Biosciences, San Diego, CA, USA). Cell Quest acquisition software (BD Biosciences) was employed to analyze the percentage of cells in different cell cycle phases.

### Flow cytometric analysis of cell apoptosis

The Annexin V/PI assay was performed based on the manufacturer’s instructions (Invitrogen). AGS cells were collected and next rinsed with cold PBS, centrifuged, and resuspended in 100 µL binding buffer containing 2.5 µL fluorescein isothiocyanate-labeled Annexin V and 1 µL 100 µg/mL PI, followed by incubation for 15 min in the dark. Afterwards, cells were detected utilizing the flow cytometer.

### Reverse transcription quantitative polymerase chain reaction (RT-qPCR)

Total RNA was separated from cultured cells using Rneasy Mini kits (Qiagen, Valencia, CA, USA). qPCR analyses were processed on a 96-well plate ABI Prism7500 System (ABI, Foster City, CA, USA) utilizing SYBR Green PCR Master Mix (Takara, Otsu, Japan). The primers used were as follows: the forward of BTG3 was 5′-CTCCTCCTGTTCCATTTGGT-3’ and the reverse was 5′-TAATCCAGTGATTCCGGTCA-3′. Cycling conditions were as follows: 5 s at 94°C, 34 s at 60°C, and 72°C for 40 cycles. Glyceraldehyde-3-phosphate dehydrogenase (GAPDH) acted as internal control. The forward of GAPDH was 5′-GTCTTACTCCTTGGAGGCC-3′ and the reverse was 5′-TCATTTCCTGGTATGACAACGA-3′. Quantitative expression was computed utilizing the 2^−ΔΔCt^ method [24].

### Ca^2+^ detection

Intracellular Ca^2+^ level was measured using the Fluo-3 AM fluorescence working solution (Beyotime, Shanghai, China). Briefly, AGS cells treated differently were collected and subsequently added with Fluo-3 AM fluorescence working solution at 37 °C for another 30 min. Cells were rinsed thrice with PBS buffer and photographs were obtained using a fluorescence microscope (magnification: 200×; Nikon, Tokyo, Japan). Intracellular Ca^2+^ concentration was monitored through flow cytometry.

### Western blot

Cells were treated with radio-immunoprecipitation assay buffer (89,901; Thermo Fisher Scientific, Waltham, MA, USA) at 4 °C to extract proteins from cells. Protein concentration was determined by bicinchoninic acid kits (Thermo Fisher Scientific). Proteins (30 µg/lane) were isolated by 10% sodium dodecyl sulfate-polyacrylamide gel electrophoresis and transferred to polyvinylidene fluoride membranes. Subsequently, membranes were incubated at 4 °C with 5% nonfat dry milk overnight and with primary antibodies against glucose-regulated protein 78 (Grp78, 1:1000, ab21685, Abcam, Cambridge, UK), C/EBP homologous protein (CHOP, 1:1000, 5554, Cell signaling technology, USA), BTG3 (1:1000, ab92309, Abcam), PI3K (1:2000; ab140307, Abcam), p-PI3K (1:1000; ab182651, Abcam), AKT (1:1000, ab8805, Abcam), p-AKT (1:1000, ab38449, Abcam), and GAPDH (1:1000, ab9485, Abcam) for 24 h. After washing with PBS-containing Tween-20, membranes were incubated for 2 h with goat anti-rabbit immunoglobulin G (1:2000, ab6721, Abcam) horseradish peroxidase-labeled secondary antibody at 4 °C. Next, enhanced chemiluminescence substrates (Generay Biotech, Shanghai, China) were used to detect the membranes.

### Animal experiments

Total 24 five-week-old male BALB/c nude mice provided by Shanghai Laboratory Animal Center (Shanghai, China) were reared in the specific-pathogen-free laboratory animal room and provided with a sterile diet under a controlled temperature of 26 ± 1 °C, humidity of 40–60%, and light exposure for 10 h/24 h. After one week of adaptive feeding, the xenograft model was established by subcutaneously injecting 100 µL AGS cells (containing approximately 1 × 10^6^ cells) into the right rib of each mouse. Three weeks later, the nude mice were intragastrically administered with 0.2 mL normal saline (control group), or baicalein at 15 mg/kg/day or 50 mg/kg/day for 4 weeks, with 8 nude mice per group. Tumor size was measured weekly after treatment and calculated as length × width × width/2. Later, 2 h after the last treatment, mice were euthanized and the tumors were removed and weighed for subsequent experiments.

### Immunohistochemical study

The formalin-fixed paraffin-embedded (FFPE) sections were subjected to immunohistochemistry. FFPE GC tumor sections were cut (3 μm), deparaffinized in xylene, and rehydrated in graded alcohols and distilled water. Endogenous peroxidase was blocked for 5 min with 3% hydrogen peroxide in distilled water. Nonspecific binding was blocked for 30 min with normal horse serum at 37 °C. Sections were subsequently incubated with Ki67 (1:500, ab16667, Abcam). Ki67 levels were detected using VETASTAIN ABC kits (Vector Laboratories, Burlingame, CA, USA).

### TUNEL assay

TUNEL assay was conducted in line with the manufacturer’s protocol (In Situ Cell Death Detection kit, POD, Roche, Basel, Switzerland). In brief, after fixing with 4% paraformaldehyde in PBS and washing thrice with PBS, cells were rinsed with 3% H_2_O_2_ in methanol for 10 min. Following incubation for 2 min with 0.1% Triton X-100 in 0.1% sodium citrate on ice, cells were then incubated for 1 h with the TUNEL reaction mixture and later with 4’,6-diamidino-2-phenylindole for 5 min in the dark. A fluorescence microscope (Eclipse 80I, Nikon) was applied for data analyses.

### Statistical analysis

All cell experiments were independently conducted in triplicate. Data were exhibited as mean ± standard deviation (SD). SPSS 21.0 software (IBM Corp. Armonk, NY, USA) was applied for data analyses. Firstly, the normality and homogeneity of variance tests were performed and the tests conformed to the normal distribution and the variances were homogeneous. The measurement data were displayed as mean ± SD. The comparisons among multiple groups were analyzed by the one-way ANOVA, followed by Tukey’s test. GraphPad Prism 8 was employed for data plotting. The *p* < 0.05 indicated statistically significant.

## Results

### Baicalein inhibited GC cell proliferation and induced G0/G1 arrest and apoptosis by regulating cell cycle

**Fig. 1:**
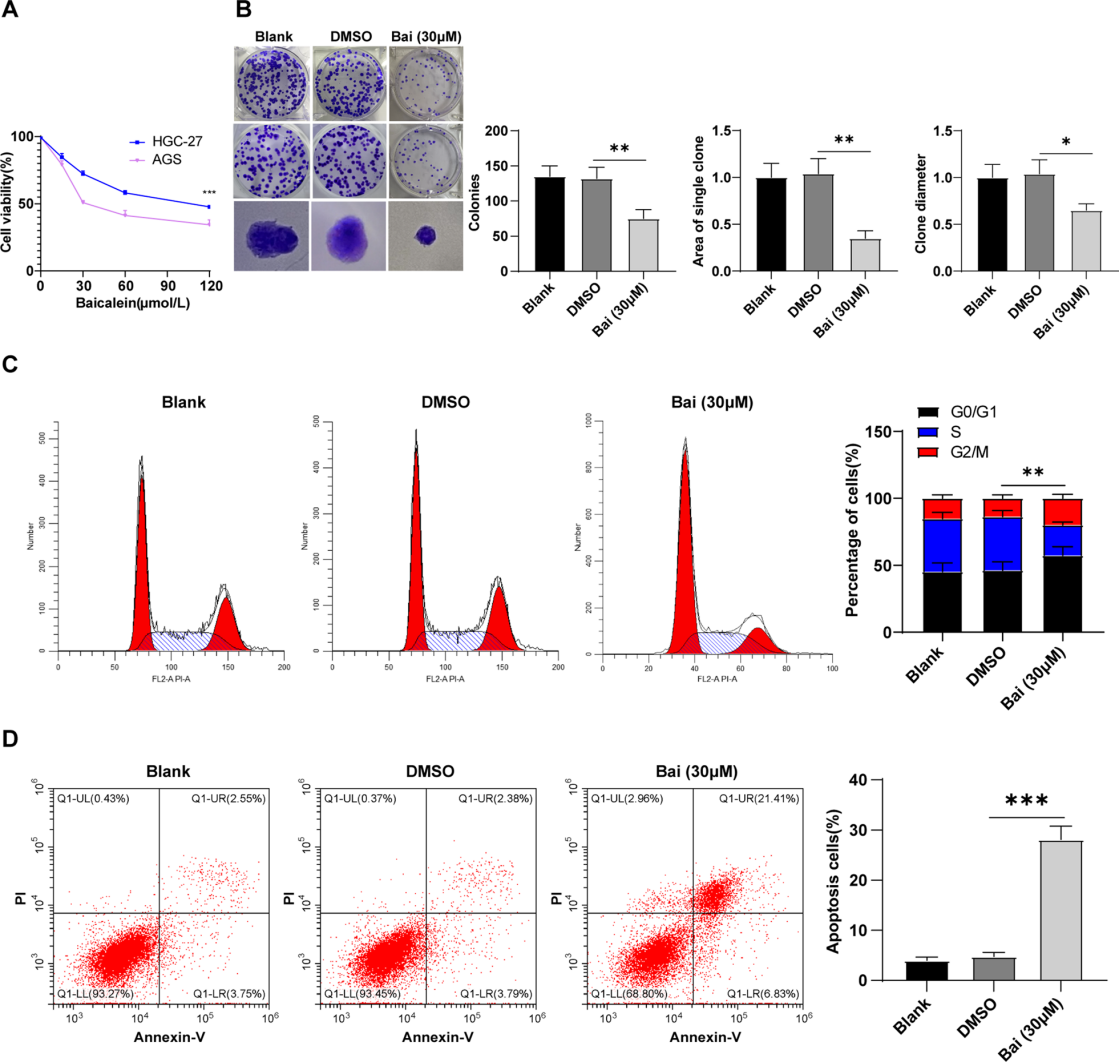
Baicalein inhibited GC cell proliferation and induced G0/G1 arrest and apoptosis by regulating cell cycle. **A** Cell viability detected by the MTT assay; **B** Colony number, colony diameter, and colony area were detected by the colony formation assay; **C** Cell cycle detected using flow cytometry; **D** Apoptosis detected using flow cytometry. Cell experiment was repeated thrice, and data were expressed as mean ± SD. One-way ANOVA was used for comparisons among multiple groups, followed by Tukey’s test. *p < 0.05, **p < 0.01, ***p < 0.001

Firstly, the action of baicalein on GC cell growth was detected by the MTT assay and GC cell lines HGC-27 and AGS were cultured using different concentrations of baicalein. We noted that baicalein effectively repressed GC cell proliferation in a concentration-dependent manner, while there was no evident difference in the suppression of 120 µM baicalein and 60 µM baicalein on GC cell viability (Fig. 1A). Moreover, the repression of baicalein on AGS cell viability was better than that of HGC-27 cells, and therefore AGS cells were used as the study subjects. The IC50 (50% inhibitory concentration) of baicalein against AGS cells was approximately 30 µM via the cell viability curve (Fig. 1A). The AGS cells were assigned into the blank group, DMSO group, and Bai (30 µM) groups. The results evinced that baicalein markedly reduced colony number, colony diameter, and colony area (Fig. 1B, all *p* < 0.05). To gain insight into the mechanism by which baicalein prevented GC cell growth, we adopted flow cytometry to detect the cell cycle of baicalein-treated AGS cells. Baicalein treatment noticeably caused more cells arrested in G0/G1 phase and fewer cells in the S phase (Fig. 1C, p < 0.05). To test whether baicalein could induce GC cell death, we detected apoptosis using flow cytometry, which indicated the promotion of baicalein in GC cell apoptosis (Fig. 1D, p < 0.001). Additionally, compared with the blank group, DMSO showed no obvious effects on AGS cells (Fig. 1B−D, all *p* > 0.05). Overall, baicalein prevented GC cell proliferation and induced G0/G1 arrest and GC cell apoptosis by mediating the cell cycle.

### Baicalein triggered ERS in GC cells

**Fig. 2:**
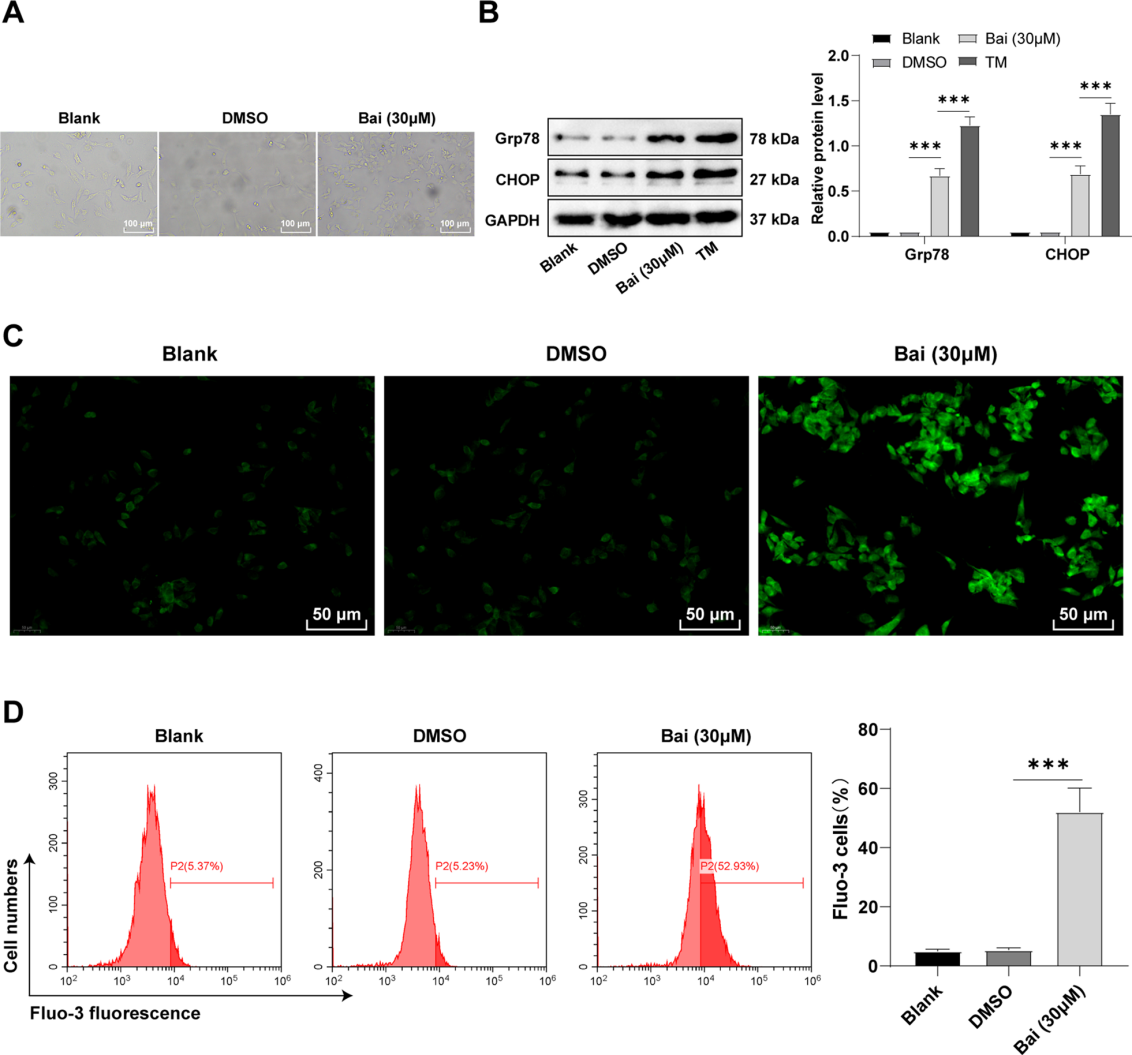
Baicalein triggered ERS in GC cells. **A** Cell morphology observed under a microscope; **B** Expression levels of ERS markers Grp78 and CHOP determined using Western blot; **C** Intracellular Ca2 + levels measured by Fluo-3 AM fluorescent probes; **D** Intercellular Ca2 + levels measured by flow cytometry. Cell experiment was duplicated thrice, and data were exhibited as mean ± SD. One-way ANOVA was adopted for comparisons among multiple groups, followed by Tukey’s test. *p < 0.05, **p < 0.01, ***p < 0.001

During baicalein-induced apoptosis, we observed the presence of cellular vacuolization in 30 µM baicalein-treated AGS cells using microscopy, but not in normal cells (Fig. 2A). Hence we speculated that these cytoplasmic vacuoles might be the dilated ER lumen under stress [25]. Therefore, we used Western blot to detect the ERS markers Grp78 and CHOP [26], with tunicamycin (TM, 5 µg/mL)-treated cells as positive controls for ERS-induced GC cells. The results demonstrated that, compared with GC cells without baicalein treatment, the expression levels of Grp78 and CHOP were increased after baicalein treatment (Fig. 2B, p < 0.05), indicating the triggering of ERS in GC cells. Cellular calcium homeostasis remains one of the functions of ER and elevated intracellular Ca^2+^ level is considered a vital indicator of ERS [27]. The results unveiled that the intracellular Ca^2+^ levels were prominently raised in baicalein-induced cells, reaching more than 10-fold higher than that of control cells (Fig. 2C, D, all *p* < 0.05). Relative to the blank group, DMSO showed no significant effects on ERS in AGS cells (Fig. 2A−D, all *p* > 0.05). Altogether, baicalein could trigger ERS in GC cells.

### Baicalein induced apoptosis by triggering ERS in GC cells

**Fig. 3:**
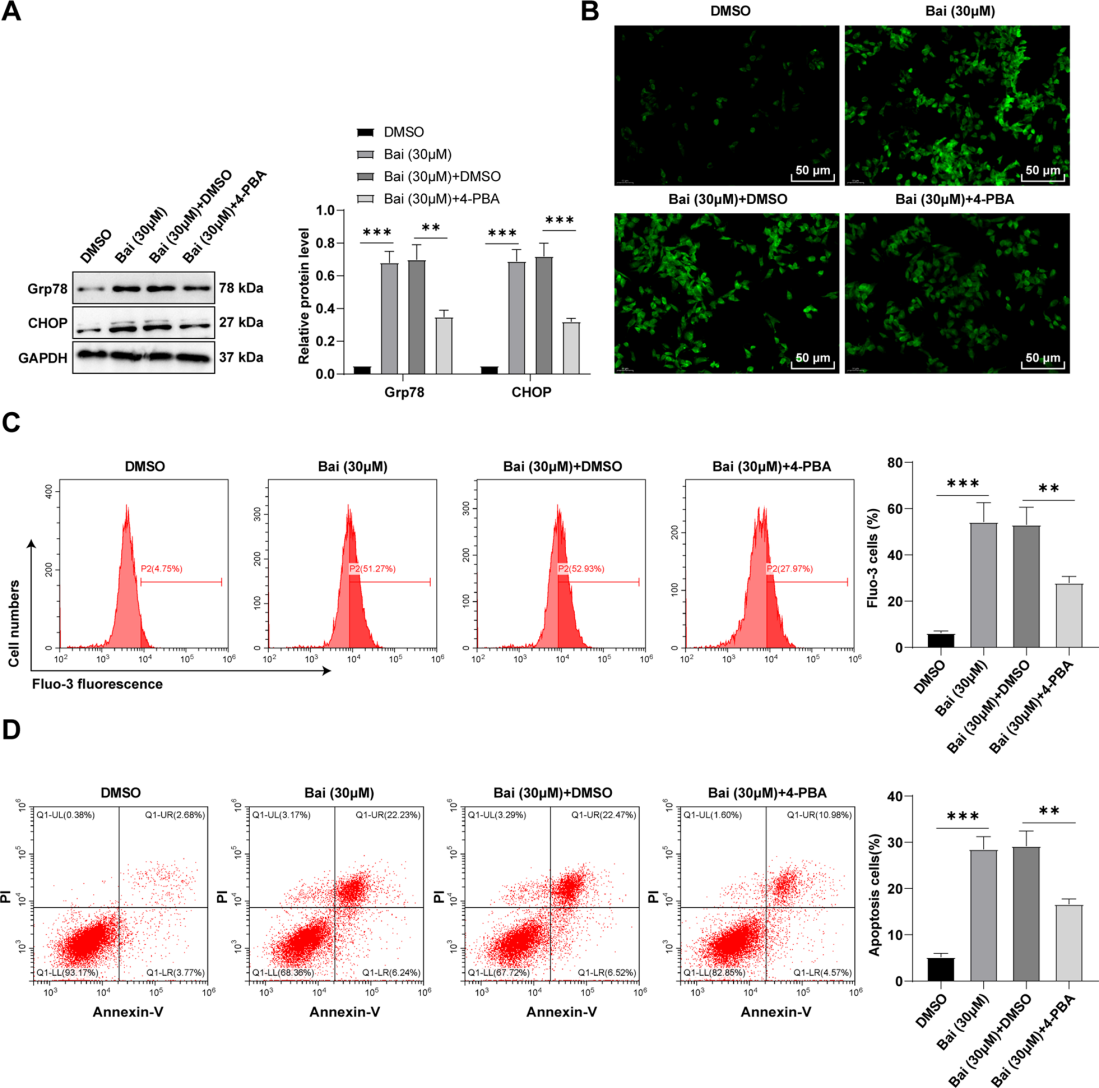
Baicalein induced apoptosis by triggering ERS in GC cells. **A** Expression levels of ERS markers Grp78 and CHOP measured using Western blot; **B** Intracellular Ca2 + levels determined by Fluo-3 AM fluorescent probes; **C** Intercellular Ca2 + levels measured by flow cytometry; **D** Cell apoptosis detected by flow cytometry. Cell experiment was replicated thrice, and the data were presented as mean ± SD. One-way ANOVA was used for comparisons among multiple groups, followed by Tukey’s test. **p < 0.01, ***p < 0.001

To assess whether baicalein-induced GC cell apoptosis was affected by ERS, we used the ERS inhibitor 4-PBA to pre-treat GC cells and subsequently treated the cells with 30 µM baicalein for 48 h. Firstly, Western blot revealed that ERS inhibitor 4-PBA partly annulled the effects of baicalein on upregulating Grp78 and CHOP levels (Fig. 3A, all *p* < 0.01). Additionally, intracellular Ca^2+^ concentration was measured by Fluo-3 AM calcium-sensitive fluorescent probes and flow cytometry, which revealed that 4-PBA partially counteracted the promotion role of baicalein in Ca^2+^ concentration (Fig. 3B, C, all *p* < 0.01). The induced ERS can trigger the UPR to respond to environmental factors. Afterwards, flow cytometry was employed to verify GC cell apoptosis, which unraveled that 4-PBA could partially reverse the promotion of baicalein on apoptosis (Fig. 3D, all *p* < 0.01). Briefly, baicalein induced apoptosis by triggering ERS in GC cells.

### Baicalein triggered ERS in GC cells to induce apoptosis by activating BTG3

**Fig. 4:**
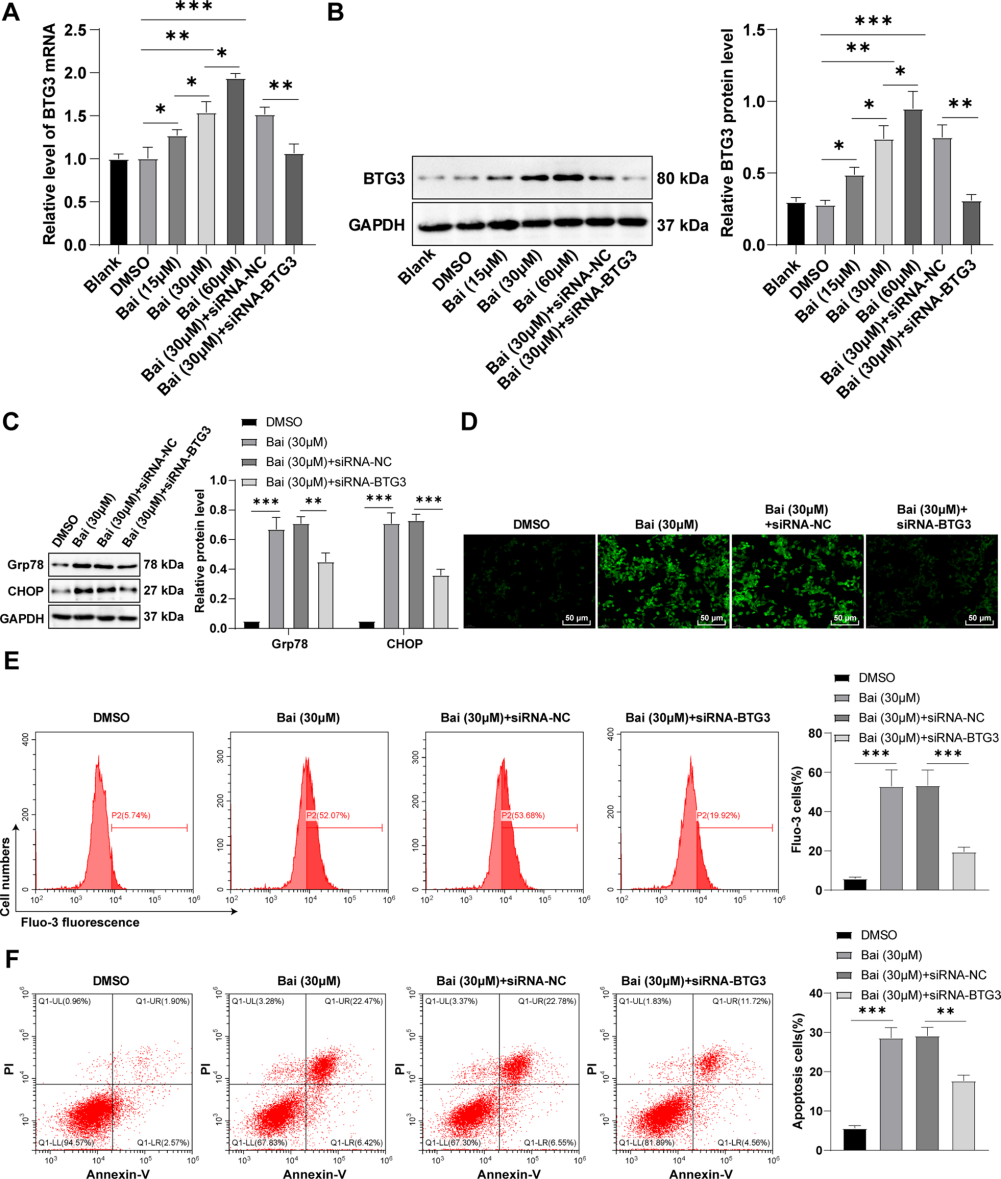
Baicalein triggered ERS in GC cells to induce apoptosis by activating BTG3. **A**, **B** BTG3 mRNA and protein expression determined by RT-qPCR and Western blot; **C** Expression levels of Grp78 and CHOP determined by Western blot; **D** Intracellular Ca2 + levels measured by Fluo-3 AM fluorescent probes; **E**: Intercellular Ca2 + levels measured by flow cytometry; **F**: Apoptosis assessed by flow cytometry. Cell experiment was repeated thrice, and the data were showed as mean ± SD. One-way ANOVA was employed for comparisons among multiple groups, followed by Tukey’s test. *p < 0.05, **p < 0.01, ***p < 0.001

BTG3, a candidate tumor suppressor, prevents cell proliferation, induces apoptosis, and mediates cell cycle in various tumors [22, 28]. Firstly, we found that BTG3 expression presented a concentration-dependent increase in GC cells under 15, 30, and 60 µM baicalein treatment (Fig. 4A, B). To investigate the role of BTG3 in baicalein-induced apoptosis, we inhibited BTG3 expression in GC cells. The expression levels of BTG3 in GC cells of each treatment group were determined by RT-qPCR and Western blot, which unveiled the successful transfection of BTG3-siRNA into GC cells (Fig. 4A, B, all *p* < 0.01). BTG3 silencing partially invalidated the facilitation effects of baicalein on Grp78, CHOP (Fig. 4C, all *p* < 0.01), and Ca^2+^ levels (Fig. 4D, E, all *p* < 0.01). Moreover, BTG3 silencing lowered the proportion of apoptotic cells and counteracted the baicalein-induced apoptosis (Fig. 4F, all *p* < 0.01). Taken together, baicalein triggered ERS in GC cells via the activation of BTG3, thus inducing apoptosis.

### Baicalein triggered ERS to induce apoptosis by impeding the PI3K/AKT pathway via the activation of BTG3

**Fig. 5:**
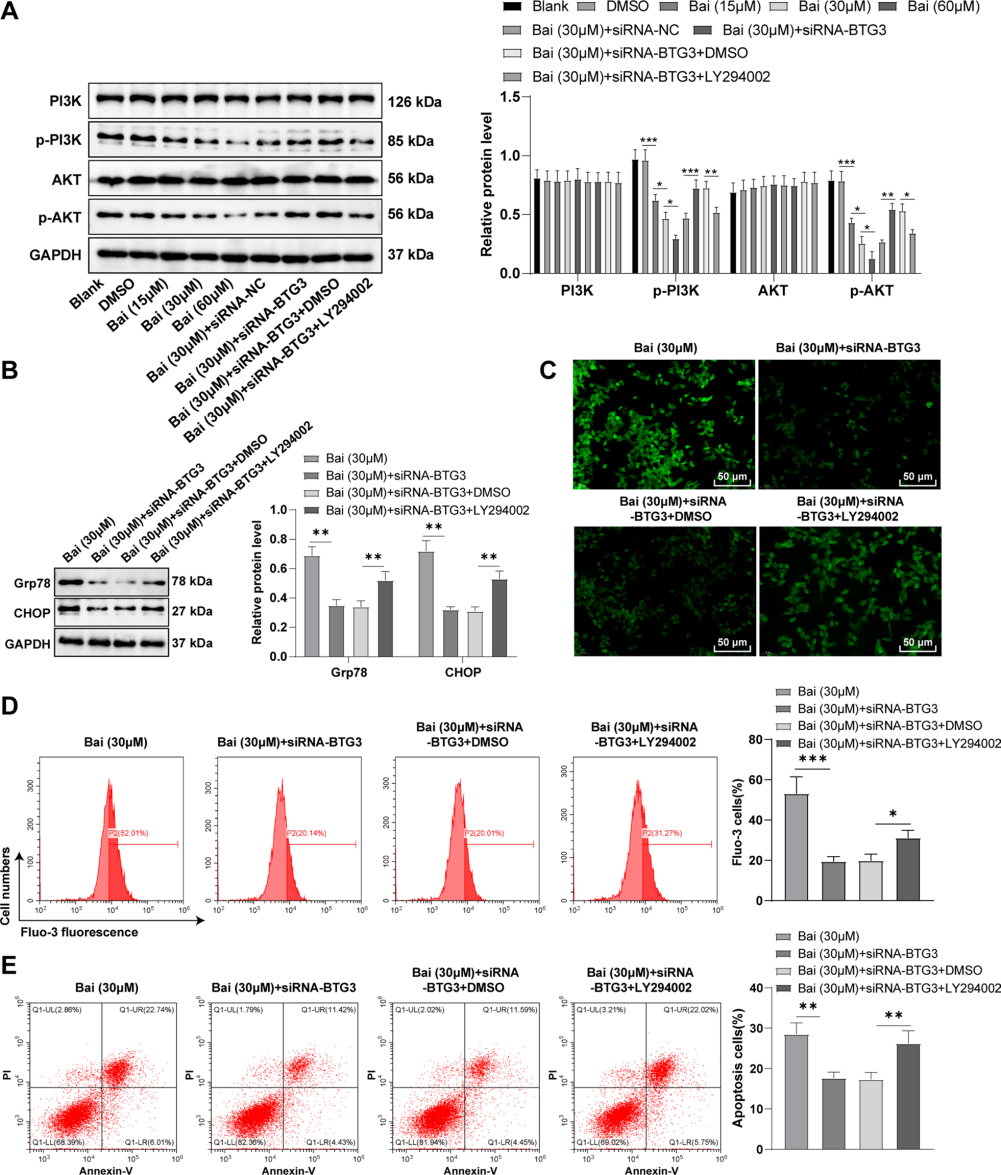
Baicalein triggered ERS to induce apoptosis by inhibiting the PI3K/AKT pathway via the activation of BTG3. **A** PI3K, p-PI3K, AKT, and p-AKT proteins determined by Western blot; **B** Expressions levels of Grp78, and CHOP determined by Western blot; **C** Intracellular Ca2 + levels measured using Fluo-3 AM fluorescent probes; **D** Intercellular Ca2 + levels measured using flow cytometry; **E** Apoptosis detected by flow cytometry. Cell experiment was duplicated thrice, and the data were expressed as mean ± SD. One-way ANOVA was used for comparisons among multiple groups, followed by Tukey’s test. *p < 0.05, **p<0.01, ***p < 0.001

PI3K/AKT is an imperative intracellular pathway; when aberrantly activated, it can activate downstream molecules, thus affecting GC development [29, 30]. Herein, we guessed that BTG3 could participate in ERS-initiated apoptosis via the PI3K/AKT pathway. Firstly, we demonstrated that after adding baicalein (15, 30, and 60 µM) to GC cells, PI3K/AKT pathway activity was inhibited and PI3K and AKT phosphorylation levels were prominently reduced in a concentration-dependent manner (Fig. 5A, all *p* < 0.05). In addition, we silenced BTG3 by cell transfection and then added 30 µM baicalein. BTG3 knockdown partially annulled the suppression of baicalein on the PI3K/AKT pathway. Subsequently, we supplemented the PI3K inhibitor (10 µM LY294002) to baicalein-treated siRNA-BTG3-transfected GC cells. Western blot results illustrated that LY294002 diminished p-PI3K and p-AKT levels in GC cells (Fig. 5A, all *p* < 0.01), indicating the blocking of the PI3K/AKT pathway. However, Grp78 and CHOP levels were noticeably elevated (Fig. 5B, all *p* < 0.01) and Ca^2+^ levels were also raised (Fig. 5C, D, all *p* < 0.05), suggesting the initiation of ERS in GC cells. Furthermore, flow cytometry revealed that LY294002 significantly increased the proportion of apoptotic cells (Fig. 5E, all *p* < 0.01). The above results demonstrated that LY294002 partly reversed the antagonistic effects of BTG3 downregulation on baicalein. In brief, baicalein inhibited the PI3K/AKT pathway by activating BTG3, thereby triggering ERS and inducing apoptosis.

### **Baicalein effectively prevented GC progression*in vivo***

**Fig. 6:**
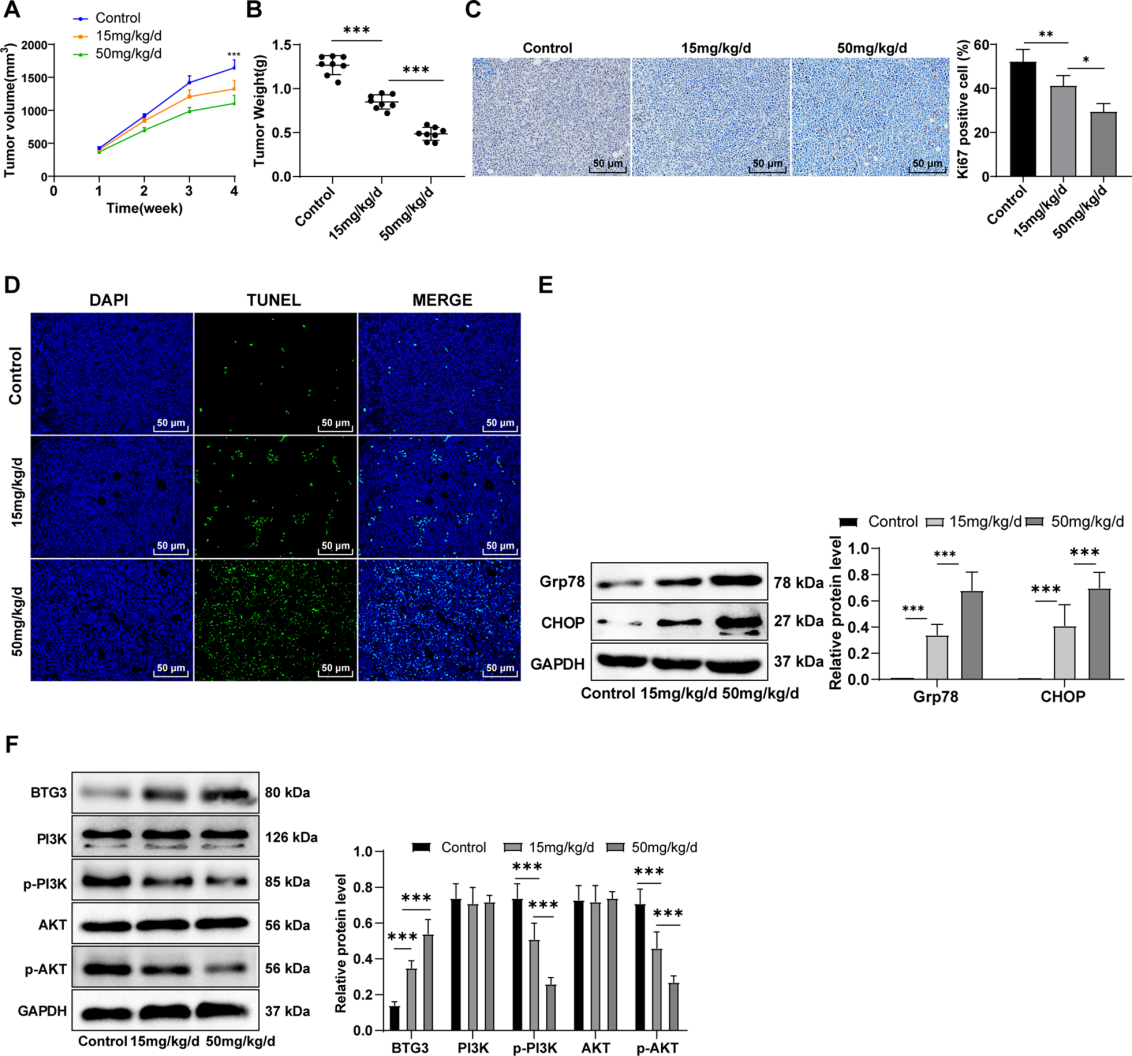
Baicalein effectively prevented GC progression in vivo. **A** Tumor volume during baicalein treatment; **B** Tumor weight after baicalein treatment; **C** Immunohistochemical staining of Ki67; **D** Apoptotic cells detected using TUNEL staining; **E**, **F** ERS-related and BTG3/PI3K/AKT pathway-related proteins detected by Western blot. The data were exhibited as mean ± SD. One-way ANOVA was adopted for comparisons among multiple groups, followed by Tukey’s test. *p<0.05, **p<0.01, ***p < 0.001

To validate whether baicalein could inhibit GC in vivo, we established subcutaneous xenograft models. The tumor growth curve revealed the repression of baicalein at 15 and 50 mg/kg/d on xenograft tumor growth (Fig. 6A, p < 0.01), and smaller tumors were observed after 50 mg/kg/d baicalein treatment. Additionally, mice in the treatment groups showed markedly lower tumor weight than the control mice (Fig. 6B, p < 0.001). The expression of Ki67 in tumors was detected by immunohistochemistry to reveal the cell proliferation in tumors, and the staining results showed that the number and staining degree of Ki67-positive cells were notably decreased upon 50 mg/kg/d baicalein treatment (Fig. 6C), indicating that baicalein treatment could relieve cell proliferation in the transplanted tumors to some extent. Moreover, TUNEL assay unveiled that the number of tumor apoptosis-positive cells was prominently higher in the 50 mg/kg/d baicalein group than in the 15 mg/kg/d baicalein and control groups (Fig. 6D). These aforesaid results indicated that baicalein effectively inhibited GC progression in vivo. To further validate the findings in vitro, after tumor homogenization, we determined the ERS-related proteins Grp78, CHOP, and the BTG3/PI3K/AKT pathway-related proteins BTG3, PI3K, AKT, p-PI3K, and p-AKT using Western blot, which revealed that BTG3 was remarkably elevated and the levels of p-PI3K and p-AKT were diminished in nude mice treated with 50 mg/kg/d baicalein compared with the 15 mg/kg/d baicalein group (Fig. 6E, F, all *p* < 0.001). Altogether, baicalein treatment triggered ERS by impeding the PI3K/AKT pathway and activating BTG3, thus inducing cell apoptosis.

## Discussion

GC represents a lethal disease with poor overall survival and most cases are attributed to diverse pathogenic infections [31]. Baicalein possesses efficient anti-tumor properties and can promote the apoptosis of different human cancer cell lines [32]. Surprisingly, tumor microenvironment stresses that break proteostasis can arise ERS, which is perceived as a trigger for apoptosis [33, 34]. Moreover, preceding evidence unravels the participation of the PI3K/AKT pathway in human GC cell autophagy and apoptosis [35]. This study demonstrated the regulation of baicalein in GC cell apoptosis by triggering ERS through suppression of the PI3K/AKT pathway.

Firstly, we treated GC cells with baicalein and found the inhibition of baicalein on cell growth. More specifically, baicalein prevented GC cell proliferation, resulted in more cells arrested in G0/G1 phases, and induced apoptosis by regulating cell cycle. Consistently, baicalein can inhibit proliferation and invasive capability, and stimulate autophagy and apoptosis in GC cells [12]. Interestingly, baicalein suppresses GC cell invasion by lowering cell migration and motility via repression of the p38 pathway [36]. Baicalein potently reduces Bcl-2 and raises Bax, which impedes colony formation and growth of GC cells and might elicit apoptosis via the mitochondrial pathway [37]. Moreover, ERS is implicated in inhibiting GC tumorigenesis via activation of Grp78 and CHOP that facilitate tumor cell growth and cell cycle arrest [38]. Likewise, Ca^2+^ is an imperative cytokine in the CaMK II pathway and can be construed as a mediator of ERS apoptosis pathway [39]. Hence, we subsequently estimated the ERS in GC cells. Our results noted that levels of Grp78 and CHOP and Ca^2+^ concentration were elevated in baicalein-treated GC cells, and the trend was partially abrogated by 4-PBA. Much in line with our finding, prior studies have noted that baicalein elicits apoptosis via Ca^2+^ generation, which also induces ERS through the Grp78 in breast cancer cells [40]. Baicalein potentiates cytosolic Ca^2+^ activity, presumably due to stimuli of cation channels in the cell membrane [41]. Additionally, baicalein induces the ERS response and up-regulation of the CHOP protein in C2C12 myotubes [42]. The above information highlighted that baicalein can trigger apoptosis and ERS in GC cells. Furthermore, to estimate whether baicalein induced GC cell apoptosis through ERS, we pre-treated GC cells using an ERS inhibitor 4-PBA, followed by baicalein treatment. Later, we observed that the ERS inhibitor nullified the promotion of baicalein on Grp78 and CHOP levels, Ca^2+^ concentration, and apoptosis. Excessive ERS contributes to apoptosis and blockage of ERS attenuated scoulerine-induced apoptosis in colorectal cancer cells [43]. Consistently, schizandrin A stimulates apoptosis and impedes GC cell growth by activating ERS [44]. Baicalein induces hepatocellular carcinoma apoptosis by triggering ERS, presumably by lowering the anti-apoptotic regulators and elevating intracellular Ca^2+^ [45]. In consistency with the aforementioned findings, our results ascertained that baicalein could induce GC cell apoptosis by initiating ERS.

Compelling evidence has unraveled the tumor-suppressive properties of BTG3 in numerous tumors: for instance, overexpression of BTG3 prevents the GC cell invasion [46]. Therefore, we determined BTG3 expression and found up-regulated BTG3 levels in baicalein-treated GC cells in a baicalein concentration-dependent manner. Afterwards, GC cells were transfected with BTG3-siRNA to explore the function of BTG3 in baicalein-elicited apoptosis. The results revealed that BTG3 knockdown noticeably diminished Grp78, CHOP, and Ca^2+^ levels, and counteracted the inducement of baicalein on apoptosis. BTG3 is weakly expressed in GC tissues and prevents GC cell growth by lowering cell-cycle progression and augmenting apoptosis [22]. Overall, baicalein triggered ERS in GC cells to induce apoptosis by activating BTG3.

GC represents one of the most influenced cancers through the PI3K/AKT pathway [47], thus arising an assumption that BTG3 is involved in the ERS-triggered apoptosis via the PI3K/AKT pathway. In our study, the phosphorylation of PI3K and AKT in GC cells was lowered after baicalein treatment in a baicalein concentration-dependent manner. Growing studies have elucidated the anti-cancer roles of baicalein by impeding the PI3K/AKT pathway in cervical cancer [48], undifferentiated thyroid cancer [49], and lung adenocarcinoma [50]. Next, we added baicalein into BTG3-downregulated GC cells and observed that BTG3 silencing abrogated the suppression of baicalein on the PI3K/AKT pathway. BTG3 overexpression suppresses the activation of the PI3K/AKT/mTOR pathway; in contrast, BTG3 silencing promotes it [21]. Afterwards, the PI3K inhibitor (LY294002) was further supplemented to these GC cells and the detection results illustrated the initiation of ERS and enhanced cell apoptosis after inhibition of the PI3K/AKT pathway. Intriguingly, p-AKT level is elevated in SGC7901 and AGS cells and GC tissues, and blockade of PI3K/AKT pathway inhibits GC metastasis and induces apoptosis [51, 52]. ERS triggers cell apoptosis in bladder cancer, ovarian cancer, and lung cancer by repressing the PI3K/AKT/mTOR pathway [23, 53, 54]. Moreover, we performed in vivo experiments, which revealed that baicalein effectively inhibited GC growth by elevating BTG3 and decreasing levels of p-PI3K and p-AKT. Collectively, baicalein blocked the PI3K/AKT pathway by activating BTG3, thus triggering ERS to induce apoptosis.

ERS can be induced by various pathological stimuli, including glucose starvation, hypoxia, and oxidative stress [55]. The ERS response has been identified to have adaptive and apoptotic pathways, and the persistent ERS could initiate cancer cell apoptosis [56]. Therein regulating ERS presumably be an anti-cancer strategy. Since it is temporarily unknown by which molecular mechanism baicalein regulates cancer cells, this study determined GC cell viability following treatment with various concentrations of baicalein, and the results unraveled that baicalein impeded the PI3K/AKT pathway, activated ERS, reduced cell viability, and facilitated apoptosis in a dose-dependent pattern. However, baicalein may cause other changes such as autophagy in GC cells by activating ERS, and these changes also exhibit some therapeutic effects on cancer cells. Future studies should examine the morphology and activity of cancer cells affected by baicalein and explore other possible alterations in cancer cells resulted from baicalein by activating ERS.

## Data Availability

All the data generated or analyzed during this study are included in this published article.
